# Isolation and Purification of Three Ecdysteroids from the Stems of *Diploclisia glaucescens* by High-Speed Countercurrent Chromatography and Their Anti-Inflammatory Activities In Vitro

**DOI:** 10.3390/molecules22081310

**Published:** 2017-08-07

**Authors:** Lei Fang, Jialian Li, Jie Zhou, Xiao Wang, Lanping Guo

**Affiliations:** 1 School of Biological Science and Technology, University of Jinan, Jinan 250022, China; fleiv@163.com; 2 Key Laboratory of TCM Quality Control Technology, Shandong Analysis and Test Center, Jinan 250014, China; jianlian_li@126.com (J.L.); wxjn1998@126.com (X.W.); 3 State Key Laboratory of Dao-di Herbs, National Resource Center for Chinese Materia Medica, China Academy of Chinese Medical Sciences, Beijing 100700, China

**Keywords:** *Diploclisia glaucescens*, ecdysteroids, high-speed counter-current chromatography, anti-inflammatory activities

## Abstract

High-speed counter-current chromatography was used to separate and purify ecdysteroids for the first time from the stems of *Diploclisia glaucescens* using a two-phase solvent system composed of ethyl acetate–*n*-butanol–ethanol–water (3:0.2:0.8:3, *v*/*v*). Three ecdysteroids were obtained from 260 mg of ethyl acetate extract of the residue obtained after evaporation of the crude ethanolicextractof *D. glaucescens* in one-step separation, which were identified as paristerone (**I**, 30.5 mg), ecdysterone (**II**, 7.2 mg), and capitasterone (**III**, 8.1 mg) by electrospray ionization mass spectrometry (ESI-MS) and nuclear magnetic resonance (NMR). Their anti-inflammatory activities were evaluated by measuring the inhibitory ratios of β-glucuronidase release in rat polymorphonuclear leukocytes (PMNs) induced by platelet-activating factor. Compounds **I**–**III** showed significant anti-inflammatory activities with IC_50_-values ranging from 1.51 to 11.68 μM, respectively.

## 1. Introduction

*Diploclisia glaucescens* (Menispermaceae) is a medicinal plant widely distributed in Guangxi and Yunnan province, China. The leaves and stems of the plant have been used as a folk medicine in the treatment of rheumatism, snake venom, biliousness, and venereal diseases [1]. Phytochemical investigations revealed that ecdysteroidsare the major components of *D. glaucescens* [2,3,4]. Ecdysteroids exhibit interesting pharmacological effects on mammals, such as the stimulation of protein synthesis, antidiabetic activity, and antirheumatic activity [5,6,7]. Furthermore, ecdysteroids are steroid hormones, that play a highly complex ecological role in the chemical communication among the kingdoms [8]. In view of these beneficial biological properties and broad applications, the study on the separation and purification of ecdysteroids from *D. glaucescens* is necessary.

**Figure 1:**
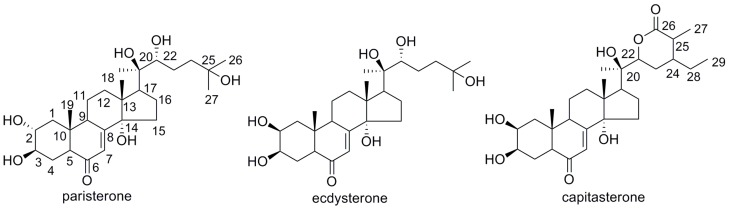
Chemical structures of three ecdysteroids from *D. glaucescens* (**I**: paristerone; **II**: ecdysterone; **III**: capitasterone).

However, the preparative separation of ecdysteroids is of significant technical challenge because plants usually produce a rather complex ecdysteroids with minor differences [9]. In the past, ecdysteroids had been isolated from *D. glaucescens* mostly based on the use of sequential chromatograpic separations on silica gel column [10,11]. Unfortunately, the conventional methods consumed large quantities of organic solvents, and often offer low recoveries of the target products. High-speed counter-current chromatography (HSCCC), being a kind of liquid–liquid partition chromatography, eliminates the irreversible adsorption of analytes on the solid phase in conventional column chromatography and offers full recovery of target compounds. It is gaining popularity in the separation and purification of different kinds of natural products [12,13]. To the best of our knowledge, few reports have been published on the separation and purification ecdysteroids from *D. glaucescens* by HSCCC. Only 20-hydroxyecdysone was successfully isolated by means of HSCCC from *Radix Serratulae Chinensis* [14]. In the present paper, an efficient HSCCC method was developed for the separation and purification of ecdysteroids from *D. glaucescens*. As a result, three ecdysteroids, paristerone (**I**), ecdysterone (**II**), and capitasterone (**III**) (Figure 1), were obtained by HSCCC from the ethyl acetate extract of the residue obtained after evaporation of the crude ethanolicextract of *D. glaucescens*. Furthermore, the anti-inflammatory activities of these three ecdysteroids were well evaluated by measuring the inhibitory ratios of β-glucuronidase release in rat polymorphonuclear leukocytes (PMNs) induced by platelet-activating factor.

## 2. Results and Discussion

### 2.1. Optimization of HPLC Conditions

**Figure 2:**
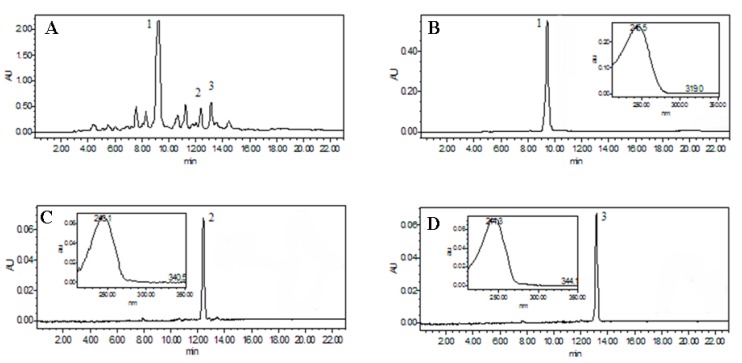
(**A**) HPLC chromatogram of the ethyl acetate extract from *D. glaucescens*; (**B**–**D**) HPLC analyses and UV spectrum of three ecdysteroids from *D. glaucescens* purified with HSCCC.

The HPLC conditions were carefully optimized, including different mobile phases with different elution modes, different detection wavelengths, and different flow rates. The results indicated that the optimized mobile phase was acetonitrile (Solvent A) and water (Solvent B) with a gradientelution (0 min 40% A; 23 min 75% A). The flow rate and detection wavelength were performed at 1.0 mL/min, and 254 nm, respectively, which were most suitable for the analysis of the crude extract. Under optimized HPLC conditions, the ethyl acetate extract of *D. glaucescens* was analyzed and presented in Figure 2. As shown in Figure 2, the extract is an extremely complex sample. In the current work, Peaks 1–3 were selected as the target for HSCCC separation.

### 2.2. Selection of HSCCC Separation Conditions

**Figure 3:**
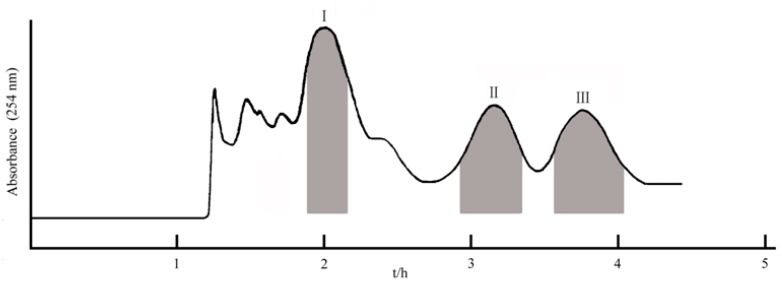
HSCCC chromatogram of the crude sample of *D. glaucescens*. Solvent system: ethyl acetate–*n*-butanol–ethanol–water (3:0.2:0.8:3, *v*/*v*); revolution speed: 850 r/min; flow rate: 2.0 mL/min; sample size: 260 mg; UV detection wavelength: 254 nm.

The choice of a suitable two-phase solvent system is the first and critical step in a HSCCC experiment. To achieve a successful separation using HSCCC, the suitable solvent system should provide an ideal range of partition coefficient (*K*, 0.5–2) for ecdysteroids and a proper separation factor (α > 1.5) between the three components. To select a suitable solvent system, several kinds of solvent systems were tested and the values of *K* for the three ecdysteroids in different solvent systems were summarized in Table 1. As shown in Table 1, the *K*-values of three ecdysteroids in the two-phase solvent systems composed of ethyl acetate-*n*-butanol–water (3:1:3, *v*/*v*) were greater than 2, which led to band broadening and excessive time for elution. While *n*-butanol in above two-phase solvent systems was replaced by ethanol, the *K*-values of three ecdysteroids decreased, especially the value of compound **I** was smaller than 0.5, which result in poor resolution of the targeted compounds. Based on the above data, the two-phase solvent systems composed of ethyl acetate–*n*-butanol–ethanol–water were tested. It can be seen that the *K*-values in the solvent systems with the volume ratio of 3:0.2:0.8:3 (*v*/*v*) were suitable for separation of the target compounds. As shown in Figure 3, good resolution and acceptable separation time could be obtained when ethyl acetate–*n*-butanol–ethanol–water (3:0.2:0.8:3, *v*/*v*) was used as the two-phase solvent system for this separation in these optimized conditions.

### 2.3. Purification of Three Ecdysteroids by HSCCC

The ethyl acetate extract (260 mg) was separated and purified in one step by the preparative HSCCC with ethyl acetate–*n*-butanol–ethanol–water (3:0.2:0.8:3, *v*/*v*) as a solvent system (Figure 3). The retention of the stationary phase was 74.0%, and the separation time was within 5 h in each separation run. The HSCCC fractions were analyzed by HPLC, and the HPLC chromatograms of the collected fractions are shown in Figure 2, respectively. The separation produced 30.5 mg of paristerone with 99.1% purity, 7.2 mg of ecdysterone with 98.3% purity, and 8.1 mg of capitasterone with 98.1% purity determined by HPLC analysis. These results demonstrate the high resolving power of HSCCC.

### 2.4. Structure Identification of the Separated Compounds

The structural identification of three ecdysteroids was carried out by ESI-MS, ^1^H-NMR, and ^13^C-NMR spectra as following:

Compound **I**: ESI-MS *m*/*z*: 503 [M + Na]^+^. ^1^H-NMR (Pyridine-*d*_5_, 600 MHz) δ: 5.76 (1H, d, *J* = 1.8 Hz, H-7), 3.94 (1H, m, H-3), 3.71 (1H, d, *J* = 2.4 Hz, H-2), 3.30 (1H, brd, *J* = 5.4 Hz, H-22), 3.13 (1H, m, H-9), 2.37 (1H, m, H-17), 2.29 (1H, m, H-5), 1.18 (3H, s, H-27), 1.17 (3H, s, H-26), 1.16 (3H, s, H-21), 1.02 (3H, s, H-19), 0.88 (3H, s, H-18). ^13^C-NMR (Pyridine-*d*_5_ 150 MHz) δ: 18.3 (C-18), 20.5 (C-19), 21.5 (C-21), 22.0 (C-16), 22.3 (C-11), 26.2 (C-4), 27.7 (C-23), 29.6 (C-26), 30.1 (C-27), 32.3 (C-15), 32.6 (C-12), 36.1 (C-9), 42.7 (C-24), 44.0 (C-10), 48.6 (C-13), 50.9 (C-17), 52.4 (C-5), 69.2 (C-2), 71.6(C-25), 74.0 (C-3), 78.2 (C-20), 78.7 (C-22), 85.4 (C-14), 122.4 (C-7), 167.5 (C-8), 206.4 (C-6). According to literature [15], compound **I** was identified as paristerone.

Compound **II**: ESI-MS *m*/*z*: 479 [M − H]^−^. ^1^H-NMR (Pyridine-*d*_5_, 600 MHz) δ: 5.63 (1H, d, *J* = 2.4 Hz, H-7), 3.78 (1H, d, *J* = 2.4 Hz, H-2), 3.66 (1H, m, H-3), 3.16 (1H, brs, H-22), 2.97 (1H, m, H-9), 2.22 (1H, m, H-17), 2.08 (1H, m, H-5), 1.05 (3H, s, H-26), 1.05 (3H, s, H-27), 1.02 (3H, s, H-21), 0.81 (3H, s, H-19), 0.73 (3H, s, H-18). ^13^C-NMR (Pyridine-*d*_5_ 150 MHz) δ: 18.4 (C-18), 21.4 (C-21), 22.1 (C-11, 16), 24.8 (C-19), 27.7 (C-23), 29.3 (C-26), 30.1 (C-27), 32.1 (C-12), 32.9 (C-15), 33.1 (C-4), 35.5(C-9), 37.6 (C-1), 39.7 (C-10), 42.8 (C-24), 50.9 (C-17), 52.1 (C-5), 68.9 (C-2), 69.2 (C-3), 71.7(C-25), 78.3 (C-20), 78.8 (C-22), 85.6(C-14), 122.5 (C-7), 167.9 (C-8), 206.8 (C-6). According to literature [11], compound **II** was identified as ecdysterone.

Compound **III**: ESI-MS *m*/*z*: 503 [M − H]^−^. ^1^H-NMR (Pyridine-*d*_5_, 600 MHz) δ: 5.64 (1H, d, *J* = 2.4 Hz, H-7), 4.11 (1H, m, H-22), 3.77 (1H, d, *J* = 2.4 Hz, H-2), 3.65 (1H, m, H-3), 3.00 (1H, brs, H-9), 2.29 (1H, m, H-17), 2.21 (1H, m, H-5), 1.11 (3H, s, H-21), 0.96 (3H, d, *J* = 6.6 Hz, H-27), 0.80 (3H, s, H-19), 0.75 (3H, t, *J* = 7.2 Hz, H-29), 0.70 (3H, s, H-18). ^13^C-NMR (Pyridine-*d*_5_ 150 MHz) δ: 10.9 (C-29), 11.6 (C-27), 17.4 (C-18), 20.2 (C-11, 16), 20.6 (C-21), 23.8 (C-19), 24.3 (C-28), 26.6 (C-23), 31.2 (C-4, 12), 33.6 (C-9), 35.8 (C-24), 36.8 (C-1), 37.2 (C-25), 38.0 (C-9), 47.2 (C-13), 48.8 (C-17), 50.1 (C-5), 67.0 (C-3), 67.2 (C-2), 75.5 (C-20), 83.8 (C-22), 84.0 (C-14), 121.5 (C-7), 164.8 (C-8), 204.0 (C-6). According to literature [16], compound **III** was identified as capitasterone.

### 2.5. Anti-Inflammatory Activities of Three Ecdysteroids

Since *D. glaucescens* has been used as a folk medicine to treat rheumatism, the ecdysteroids may be possess potent anti-inflammatory activities. To confirm this hyphotesis, the anti-inflammatory activities of the three ecdysteroids have been evaluated herein by measuring the inhibitory ratios of β-glucuronidase release in rat polymorphonuclear leukocytes (PMNs) induced by platelet-activating factor. As shown in Table 2, compounds **I**–**III** showed significant anti-inflammatory activities with IC_50_-values ranging from 1.51 to 11.68 μM, respectively. Compound **I** exerts a more potent anti-inflammatory activity than the positive control used. Furthermore, compound II showed a similar (but slightly less potent) activity than the selected positive control. These obtained results suggest that the evaluated ecdysteroids can be the compounds responsible for the antirheumatic activity of this herb in tradicional medicine.

## 3. Materials and Methods

### 3.1. Apparatus

HSCCC was performed on a GS10A-2 (Beijing Emilion Science & Technology Co., Beijing, China), with a multilayer coil of 1.6 mm I.D. and 110 m in length with a total capacity of 230 mL. The β-values of this preparative column range from 0.5 at internal to 0.8 at the external (β = r/R, where r is the rotation radius or the distance from the coil to the holder shaft, and R is the revolution radius or the distances between the holder axis and central axis of the centrifuge). The HSCCC systems was equipped with a Model NS-1007 constant-flow pump, a Model 8823A-UV Monitor operating at 254 nm, a Model 320 pH meter, and a Yokogawa Model 3057 portable recorder.

HPLC was carried out by a Waters Empower system (Milford, MA, USA) coupled with a Model 600 pump, a Model 600 multi-solvent delivery system, a Model 996 diode-array detector (DAD), and an Empower workstation. The ESI-MS analyses were performed on an Agilent 1100/MSG1946 (Agilent, CA, USA). The NMR data was recorded on a Varian 600 MHz NMR spectrometer (Varian, Palo Alto, CA, USA).

### 3.2. Reagents and Materials

Analytical grade solvents used for HSCCC were purchased from Tianjing Chemical Factory (Tianjing, China). Acetonitrile used for HPLC was chromatographic grade and purchased from Siyou Special Reagent Factory (Tianjin, China). All aqueous solutions were prepared with pure water produced by a Milli-Q system (Millipore, MA, USA).

The stems of *D. glaucescens* were collected in Guangxi, China, and identified by Dr. Jia Li (College of Pharmacy, Shandong University of Traditional Chinese Medicine).

### 3.3. Preparation of Crude Sample

The stems of *D. glaucescens* (2.0 kg) were powdered and extracted three times (each for 2 h) with 95% ethanol (3 × 15.0 L) under reflux. After filtration, the extract was combined and evaporated in vacuo to afford a concentrated EtOH extract (120 g). Then, the crude extract was suspended in water (500 mL) and successively extracted with *n*-hexane (3 × 500 mL), ethyl acetate (3 × 500 mL), and *n*-butanol (3 × 500 mL). The dried ethyl acetate extract (19 g) was stored in a refrigerator for subsequent HPLC analysis and HSCCC separation.

### 3.4. Selection of Two-Phase Solvent System

The selection of two-phase solvent system is the crucial point for a successful separation by HSCCC. The solvent system was selected according to the partition coefficient (*K*) of target compound. The *K*-values were determined by HPLC as follows: Approximately 2 mg of crude extract was added to the test tube, to which 2 mL of each phase of the two-phase solvent system was added. The test tube was shaken violently for several minutes. Then, an equal volume of each phase was analyzed by HPLC to obtain the partition coefficients. The *K*-value was expressed as the peak area of the compound in the upper phase divided by that in the lower phase.

### 3.5. HSCCC Separation Procedure

In the present study, the HSCCC experiments were performed with a two-phase solvent system of ethyl acetate–*n*-butanol–ethanol–water (3:0.2:0.8:3, *v*/*v*). The solvent mixture was thoroughly equilibrated in a separation funnel by repeatedly vigorously shaking at room temperature. The two phases were separated shortly prior to use. The upper phase was used as the stationary phase, while the lower phase was used as the mobile phase. The sample solution was prepared by dissolving the dried extract in the mixture solution of lower phase and upper phase (1:1, *v*/*v*) of the solvent system.

HSCCC separation was performed as follows: the multiplayer coiled column was first filled entirely with the upper organic phase as the stationary phase. The lower aqueous phase was then pumped into the head end of the column at a suitable flowrate of 2 mL/min, while the apparatus was rotated at a speed of 850 rpm. After the hydrodynamic equilibrium was reached, as indicated by a clear mobile phase eluting from the tail outlet, the sample solution was injected into the column through the inject valve. The effluent of the column was continuously monitored with a UV detector at 254 nm and the chromatogram was recorded. Each peak fraction was collected according to the elution profile and determined by HPLC. After the separation was completed, retention of the stationary phase was measured by collecting the column contents by forcing them out of the column with pressurized nitrogen gas.

### 3.6. HPLC Analysis and Identification of HSCCC Fractions

The ethyl acetate extract of the residue obtained after evaporation of the crude ethanolic extract of *D. glaucescens* and the fractions from the preparative HSCCC separation were analyzed by HPLC. Chromatographic analysis were accomplished with anInertsil-ODS-SP column (250 mm × 4.6 mm, 5 μm) at room temperature. The mobile phase was acetonitrile (Solvent A) and water (Solvent B) with the gradient (0 min 40% A; 23 min 75% A) and performed at a flowrate of 1.0 m/min. The effluent was monitored at 254 nm by a photodiode array detector.

The identification of the HSCCC peak fractions was carried out by electrospray ionization mass spectrometry (ESI-MS) on an Agilent 1100/MS-G1946 (Agilent, Santa Clara, CA, USA) and ^1^H- and ^13^C-NMR spectra on a Varian-600NMR spectrometer (Varian, Palo Alto, CA, USA).

### 3.7. Assay for Anti-Inflammatory Activity

In the present study, the anti-inflammatory activities of the three ecdysteroids were evaluated by measuring the inhibitory ratio of β-glucuronidase release in rat PMNs induced by PAF in vitro [17]. Briefly, the samples were dissolved in DMSO at a concentration of 0.1 M and diluted to 10^−3^ M. The samples (5.0 μL) were incubated with a suspension of rat PMNs (250 μL) at a density of 2.5 × 10^6^ cells mL^−1^ at 37 °C for 15 min, and 1 × 10^−3^ M cytochalasin B (2.5 μL) were added for another 5 min. After the addition of PAF (2.5 μL), the mixture was incubated for another 5 min. Subsequently, the mixture was put in an ice-bath to terminate the reaction. After centrifugation at 4000 rpm for 5 min, the supernatant was obtained. The supernatant (25 μL) and 2.5 mM phenolphthalein glucuronic acid (25 μL) were incubated with 0.1 M HOAc buffer (100 μL) at 37 °C for 18 h. The reaction was terminated by the addition of 0.3 M NaOH (150 μL). The absorbance was read at 550 nm and the inhibitory ratio (IR) was calculated as IR (%) = (A_PAF_ − A_t_)/(A_PAF_ − A_C_) ×100%, where A_PAF_, A_t_, and A_C_ refer to the cell level of PAF, test compounds, and control groups, respectively. Ginkgolide B was used as a positive control.

## 4. Conclusions

An efficient HSCCC method was developed and successfully applied to the separation and purification of three ecdysteroids from ethyl acetate extract of the residue obtained after evaporation of the crude ethanolic extract of *D. glaucescens* using ethyl acetate–*n*-butanol–ethanol–water (3:0.2:0.8:3, *v*/*v*) as the two-phase solvent system. Three ecdysteroids, paristerone, ecdysterone, and capitasterone, showed significant anti-inflammatory activities by measuring the inhibitory ratios of β-glucuronidase release in rat polymorphonuclear leukocytes (PMNs) induced by platelet-activating factor. The results indicated that HSCCC is a suitable and effective protocol for separation of active ecdysteroids from complex mixtures extracted from natural herbs.

## Figures and Tables

**Table 1:** The *K*-values of the target compounds in several solvent system.

Solvent System (*v*/*v*)	*K*		
	I	II	III
ethyl acetate–*n*-butanol–water (3:1:3)	2.83	6.12	10.25
ethyl acetate–ethanol–water (3:1:3)	0.44	0.70	0.96
ethyl acetate–*n*-butanol–ethanol–water (3:0.4:0.6:3)	1.06	2.96	4.08
ethyl acetate–*n*-butanol–ethanol–water (3:0.3:0.7:3)	0.93	1.77	2.65
ethyl acetate–*n*-butanol–ethanol–water (3:0.2:0.8:3)	0.76	1.41	2.20

**Table 2:** Anti-inflammatory Activity of Compounds **I**–**III**.

Compounds *^a^*	IC_50_-Values (μM)
**I**	1.51
**II**	2.62
**III**	11.68
**Ginkgolide B *^a^***	2.35

*^a^* Positive control.
